# Neural Mechanisms Involved in Mental Imagery of Slip-Perturbation While Walking: A Preliminary fMRI Study

**DOI:** 10.3389/fnbeh.2018.00203

**Published:** 2018-09-26

**Authors:** Tanvi Bhatt, Prakruti Patel, Shamali Dusane, Sophie R. DelDonno, Scott A. Langenecker

**Affiliations:** ^1^Department of Physical Therapy, College of Applied Health Sciences, Chicago, IL, United States; ^2^Department of Psychiatry, College of Medicine, University of Illinois at Chicago, Chicago, IL, United States

**Keywords:** reactive balance control, locomotion, falls, imaging, neural activation

## Abstract

**Background:** Behavioral evidence for cortical involvement in reactive balance control in response to environmental perturbation is established, however, the neural correlates are not known. This study aimed to examine the neural mechanisms involved in reactive balance control for recovery from slip-like perturbations using mental imagery and to evaluate the difference in activation patterns between imagined and observed slipping.

**Methods:** Ten healthy young participants after an exposure to regular walking and slip-perturbation trial on a treadmill, performed mental imagery and observation tasks in the MR scanner. Participants received verbal instructions to imagine walking (IW), observe walking (OW), imagine slipping (IS) and observe slipping (OS) while walking.

**Results:** Analysis using general linear model showed increased activation during IS versus IW condition in precentral gyrus, middle frontal gyrus, superior, middle and transverse temporal gyrus, parahippocampal gyrus, cingulate gyrus, insula, pulvinar nucleus of the thalamus, pons, anterior and posterior cerebellar lobes. During IS versus OS condition, there was additional activation in parahippocampus, cingulate gyrus, inferior parietal lobule, superior temporal, middle and inferior frontal gyrus.

**Conclusion:** The findings of the current study support involvement of higher cortical and subcortical structures in reactive balance control. Greater activation during slipping could be attributed to the complexity of the sensorimotor task and increased demands to maintain postural stability during slipping as compared with regular walking. Furthermore, our findings suggest that mental imagery of slipping recruited greater neural substrates rather than observation of slipping, possibly due to increased sensory, cognitive and perceptual processing demands.

**New and Noteworthy:** The behavioral factors contributing to falls from external perturbations while walking are better understood than neural mechanisms underlying the behavioral response. This study examines the neural activation pattern associated with reactive balance control during slip-like perturbations while walking through an fMRI paradigm. This study identified specific neural mechanisms involved in complex postural movements during sudden perturbations, to particularly determine the role of cortical structures in reactive balance control. It further highlights the specific differences in neural structures involved in regular unperturbed versus perturbed walking.

## Introduction

About 60 percent of outdoor falls sustained by community-living older adults are due to environmental perturbations in the form of a slip or a trip (Luukinen et al., 2000). Behavioral studies exposing older adults to perturbations during walking have established several biomechanical factors such as deficient limb support, inefficient compensatory stepping, reduced stability and lower hip height as predictors of falls (Pavol et al., 2004; Pavol and Pai, 2007; Yang et al., 2009, 2011). While biomechanical causes of falls are better understood, the exact neural mechanisms and substrates involved in recovering balance from sudden falls, in other words for reactive balance control, are still not known. Considering that falls are a significant health concern in the aging and neurological population, understanding the specific neural mechanisms involved in falls occurring from sudden balance loss could assist in developing targeted interventions (Luukinen et al., 2000).

Humans rely on two main strategies for balance control and fall prevention: proactive (occurring before balance disturbance) and reactive (occurring after balance disturbance) control strategies. Proactive strategies are employed to enhance stability to prevent and compensate for a loss of balance even before it occurs (Tang et al., 1998; Ferber et al., 2002; Horak, 2006; Shumway-Cook and Woollacott, 2007). For proactive adjustments, the nervous system has adequate time to predict the upcoming balance requirements and make necessary adjustments to minimize the intensity of balance loss or prevent it altogether (Massion, 1994; Aruin and Latash, 1995). A good example is early detection and avoidance of an upcoming obstacle in the walking path (Woollacott and Tang, 1997). Proactive adjustments during locomotion and functional tasks seem to involve cortical and sub-cortical structures of the CNS for planning and execution of an appropriate motor response in anticipation of a perceived threat to balance (Armstrong and Drew, 1985; Drew, 1988; Beloozerova and Sirota, 1993; Petersen et al., 2012). Indeed, a few electrophysiological studies in humans and cats have confirmed the involvement of motor cortex in regulating accurate muscle activity and modulating step cycle when a change in the environment or obstacle in the path is perceived, (Armstrong and Drew, 1985; Drew, 1988; Beloozerova and Sirota, 1993; Petersen et al., 2012).

Unlike proactive adjustments, reactive balance strategies are the key defense mechanisms to prevent a fall after a sudden unexpected perturbation. It involves compensatory postural adjustments initiated by feedback processes (Maki and McIlroy, 1997). Overall, it is proposed that the CNS uses an internal representation of body’s mechanics to predict and identify loss of balance. Often, large postural responses displacing center of mass (COM) outside of the base of support (BOS) require change-in-support i.e., stepping to prevent a fall (Maki and McIlroy, 1997; Pai and Bhatt, 2007). Several electrophysiological studies in animals and humans support that polysynaptic spinal cord reflexes (short- and long-latency) are responsible for generating the postural response to correct or restore balance during minor disturbances that do not truly pose a threat to the person (Forssberg et al., 1975; Berger et al., 1984; Dietz et al., 1986; Diener et al., 1988; Allum and Honegger, 1992; Zehr et al., 1997). Reactive responses to restore balance from a large disturbance on the other hand, are usually controlled by supra-spinal structures in the brainstem and its descending pathways (long-loop reflexes)(Forssberg et al., 1975; Nashner, 1976, 1980; Eng et al., 1994), with some earlier evidence suggesting modulation from cortical structures (Forssberg et al., 1975). Specifically, cerebral cortex perhaps modifies postural response through cerebellar-cortical loop which helps in using prior experience and basal ganglia-cortical connections which incorporates sensory information based on the current context (Graydon et al., 2005; Jacobs and Horak, 2007).

Several studies have attempted to examine neural mechanisms of compensatory postural responses during stance perturbations using techniques such as electroencephalography (EEG) and functional near infrared spectroscopy (fNIRS) (Quant et al., 2004; Adkin et al., 2006; Mochizuki et al., 2009). However, neural substrates involved in maintaining/recovering balance during locomotor tasks have not been sufficiently examined. This could be partly related to the technical challenges that present with using functional neuroimaging such as fNIRS and EEG measures with walking in the form of either low spatial resolution or noise and artifact (Sipp et al., 2013). Although direct evidence via functional magnetic resonance imaging (fMRI) is difficult to obtain, mental imagery has been widely used to examine locomotor-balance tasks that cannot be performed in the MRI scanner such as walking (Jahn et al., 2004, 2008; van der Meulen et al., 2014) or obstacle crossing (Wang et al., 2009). Use of mental imagery is based on the premise that overlapping neural networks are engaged in both mental imagery and overt movements (Decety and Jeannerod, 1995; Porro et al., 1996). Similarly, action observation has also been used to elicit simulation of motor tasks during neuroimaging – especially gross whole body tasks that cannot be performed in the scanner (Dalla Volta et al., 2015). Action observation is believed to stimulate the neural presentation of the movement being observed through the mirror neuron system (Buccino, 2014). While there are a few studies directly comparing the cortical activation between action observation and actual movement performance, there is only one study directly comparing differences in neural activation during observed versus imagined walking (Iseki et al., 2008).

Mental imagery and action observation are increasingly used as rehabilitation tools for inducing motor learning (Morganti et al., 2003; Franceschini et al., 2010; Zhu et al., 2015; Caligiore et al., 2017). Only recently these techniques have been implemented for physical rehabilitation in clinical populations (Ietswaart et al., 2011; Hong et al., 2012; Garcia Carrasco and Aboitiz Cantalapiedra, 2016; Patel et al., 2017); however, they have been widely used to improve performance in sports (Mulder, 2007). It is believed that motor imagery and action observation can prime the CNS to enhance motor learning (fewer repetitions or lesser time) when performed before the actual motor task (Edwards et al., 2003; Hardwick and Edwards, 2011; Saimpont et al., 2013). Such learning mechanisms might be beneficial for frail or neurological populations for whom tolerability of training dosage or safety might be a concern. In the context of reactive balance control, a previous study has examined if observational learning could substitute for motor learning induced via slip perturbation-training for lowering fall-risk (Bhatt and Pai, 2008). The study did demonstrate evidence of improved postural stability with observational training although not to the same extent as that induced by actual motor training. It remains to be determined if mental imagery could have produced a similar or better effect than observational learning, especially due to animation of sensory systems when performing mental imagery with eyes closed (Marx et al., 2003).

Due to lack of literature comparing motor imagery and action observation directly, there is limited understanding of the neural structures underlying these paradigms. In the present pilot study, a novel paradigm using imagined slipping during walking was used to identify neural correlates of modulating locomotor-balance control during environmental perturbations. Our primary objective was to identify the neural networks involved in reactive balance control in response to large slip-like perturbations during walking as compared with regular walking through metal imagery. Considering that slipping while walking demands greater postural control to regain balance, we postulate that during imagined slipping while walking there will be higher activation in the frontal, parietal regions and cerebellum as compared with imagined walking. We also aimed to examine whether or not imagination of slipping while walking results in activation of different brain areas as compared to observation of the same movement. This understanding of neural correlates involved in falls may provide insights for designing observational learning or mental imagery paradigms for fall prevention, especially for populations that might not be able to directly withstand the actual task-specific perturbation training.

## Materials and Methods

### Subjects

Ten young healthy adults (26.90 ± 4.25 years) participated in the study after providing an informed consent approved by the Institutional Review Board. The subjects were screened for any systemic disorders and *fMRI* safety, and had normal vision and hearing. All the subjects then performed the experimental tasks outside and then in the MRI scanner as described below.

### Walking and Slipping on Treadmill

**FIGURE 1:**
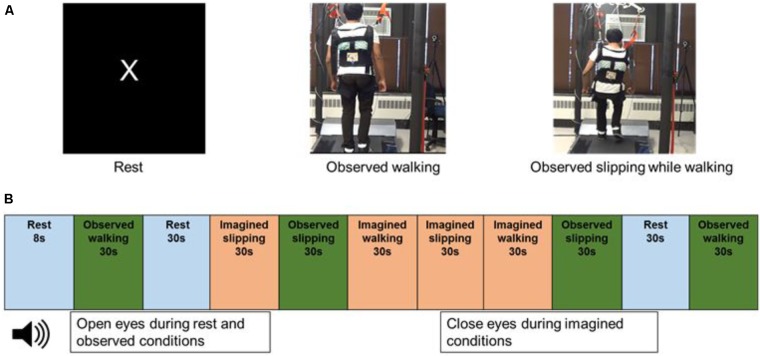
**(A)** Still images from the video displayed to the subjects while in the scanner, during rest, observed walking and observed slipping while walking. Subject has provided written informed consent for usage of recorded video for data collection during fMRI and images for publication purposes. **(B)** Order of the rest, observed and imagined conditions used in the fMRI paradigm.

All subjects were initially exposed to walking on the treadmill at a self-selected pace and then were exposed to a single slip-like perturbation with an acceleration of 12.00 m/s^2^, displacement 0.25 m and duration of 0.25 s. For these tasks, subject stood on the ActiveStep treadmill (Simbex, NH) with a comfortable stance. A harness suspended from the overhead arch was donned to prevent individuals from falling (**Figure 1A**). The treadmill belt speed was increased slowly until the individual perceived the speed to be similar to his/her regular walking speed. At this speed, the individual walked for a minute. Following regular walking, the individual performed another walking trial. During this trial individuals were warned that they may experience a slip-like perturbation after a few steps of walking, however, they were unaware of the exact instance of slip occurrence. Upon a slip-like perturbation, individuals were asked to execute their natural response to recover balance and prevent themselves from falling. The subjects were exposed to the treadmill slip before the *fMRI* experiment to provide them with an experience of how a real-life slip would feel like, especially for those people who might not have experienced a slip in recent times. Such experience could also aid in reducing within-subject variance of the observation or imagination tasks, and hence, enhance ability of the subjects to better relate to the conditions.

### Observed and Imagined fMRI Conditions

After the initial walking and slip exposure, and prior to the MRI scans, subjects underwent a practice session involving motor imagery and observation tasks. The motor imagery task comprised of (a) imagining themselves walking on the treadmill (IW) and (b) imagining themselves slipping on the treadmill while walking, similar to that experienced during initial exposure (IS). Similarly, the observation tasks consisted of (a) observing a video of another person walking on the treadmill (OW) and (b) observing a video of another person slipping while walking on the treadmill (OS) (**Figure 1A**). During the experiments, the subjects lied down supine in the scanner and the stimuli were projected on the screen in front of them through a mirror with an angle of 9° from the vertical. For the imagined conditions, subjects received clear instructions to close their eyes and that they should imagine themselves slipping where the leg suddenly slides/moves forward with respect to their body, similar to what they experienced on the treadmill. Such experimental design was purposefully chosen based on the practical realities of the research question, for example if eyes are open for imagined condition this would not be representative of a real life task (challenge to ecological validity). Furthermore, eyes closed during imagination has shown increased cortical activation of various sensory systems as the subjects recall their sensory experience from the perception of the actual activity (Marx et al., 2003). For the observed condition, subjects were instructed to observe the video of a person either walking on the treadmill or slipping while walking on the treadmill, a situation similar to what they had experienced in the laboratory. To ensure subjects engaged themselves during observation from the first person perspective, they were shown videos of back view of walking and slipping, compared with a side view. This was based on the results from a pilot study conducted on a separate set of ten young subjects, which concluded that 90% of subjects found the back view to be most engaging to view themselves slipping as compared with the side view. The mental imagery of tasks was assessed using the Vividness of Visual Imagery Questionnaire. Following familiarization to the observed and motor imagery tasks, subjects performed these tasks in the MRI scanner. All subjects reported no difficulty in performing mental imagery as indicated by their median score of 4.5/5 on the vividness of visual imagery questionnaire.

### Experimental Protocol

During the MRI scan, subjects were provided with a headset to listen to the instructions and were asked to lie still. Subjects received instructions for the imagined tasks through the headset and a screen in front of them in the scanner projecting the videos during the observed conditions. Four trials for each of the imagined and observed conditions (IW, IS, OW, and OS) were performed in the scanner. The duration of each trial was 30 s. The experimental tasks were interspersed with four trials of “Rest” condition. During the Rest condition, the screen projected the letter “X” on a black background and subjects were instructed to focus on the letter on the screen, trying not to think about anything else. The Rest condition was included to record the resting brain activity in the absence of observed or imagined tasks. The observed, imagined and rest trials were presented in a randomized order (**Figure 1B**). All subjects performed the trials in the same randomized order. Two blocks of two trials each were presented for each condition.

### Data Acquisition

Whole brain imaging was performed with a 3.0 T GE Discovery scanner (Milwaukee, WI, United States) using a standard radio frequency coil and T2^∗^-weighted pulse sequence. BOLD functional images were collected using a gradient-echo axial echo planar imaging sequence (Glover and Thomason, 2004) at UIC. The following parameters were used: repetition time = 2,000 m, echo time = 22.2 ms, flip angle = 90 degree, field of view = 22 cm, 64 by 64 matrix, slice thickness = 3 mm, 44 slices. An axial T1 SPGR structural image was obtained for each using 182 axial images 1 mm in thickness for spatial normalization [minimum TR/TE (9.292 ms/3.77 ms) TI = 450 ms]. During scanning, subjects completed the observed and imagined tasks. Prior to scanning, the importance of remaining motionless was conveyed to each subject. There were two runs of the experimental tasks, each lasting 5 min and 20 s, and acquiring 120 volumes. Each condition was averaged across both runs, prior to the creation of subtraction contrast analyses. The same scanner and acquisition sequence was used for all subjects and there was no relationship between the year the fMRI was performed and extracted activation of the BOLD signal.

### Data Processing

Preprocessing of fMRI data was conducted using SPM8^1^, FSL, and AFNI^2^. Data were despiked using AFNI. All data was then slice-time corrected in SPM8 and realigned in FSL^3^ using MCFLIRT (Jenkinson et al., 2002). Anatomical and functional images were coregistered and normalized to Montreal Neurological Institute (MNI) space using SPM8. Smoothing was completed with a full width at half maximum filter of 5 mm. First level models were built in SPM8 using x, y, and z translation realignment movement regressors from FSL for each run. The subtraction method was used to create contrast images and second level models were built in SPM8.

### Statistical Analysis

A gray matter mask was applied and whole brain correction was achieved for the multiple regression model at *p* = 0.01 by conducting 1000 Monte Carlo simulations in 3dClustSim to determine a joint threshold of height and extent (*p* < 0.005, extent of 440 mm^3^ or K = 55 voxels). The Monte Carlo approach was intended to balance Type I and Type II error and cluster extent was determined using the bug-fixed 3dClustSim tool (Cox et al., 2017). Further, the fMRI data was evaluated with planned *t*-test contrasts between selected conditions. Given the above rationales, the *p*-value was not further adjusted for multiple comparisons. We first built contrasts for each active condition by subtracting rest activation from the condition, for example: IW-minus-rest. Then, we compared activation between different conditions using contrasts created relative to the rest condition. For example, we subtracted IW-minus-rest activation from IS-minus-rest activation. In total, the following planned contrasts were used: IW-rest, IS-Rest, IS-IW, IW-OW, IS-OS.

## Results

### Mental Imagery of Walking and Slipping Versus Rest

As compared with rest condition (baseline), the cerebral activity was greater in both mental imagery conditions (walking and slipping) (**Tables 1**, **2**). Mental imagery of walking showed increased activity in the frontal lobe within the left supplementary motor area (BA 32). The imagined slipping condition resulted in a significantly greater activity in both cortical and subcortical regions as compared with the rest condition. The cortical areas included left supplementary motor areas (BA6), left superior frontal gyrus, right pars opercularis (BA45), left inferior parietal lobule (BA 40), right parahippocampal gyrus, left cingulate gyrus and right frontal inferior operculum (BA45). Among the sub-cortical structures, bilateral posterior cerebellum showed increased activation during imagined slipping.

**Table 1:** Differences in activation between imagined slipping and rest conditions.

Contrast	Anatomical Regions				mm^3^	Voxels	MNI coordinates			
	Lobe	Gyrus	BA	Side			*x*	*y*	*z*	Z-value
IS>Rest	Frontal	Precentral Frontal inferior operculum	–	L	6,112	764	–48	8	14	3.87
		Superior frontal	6	L	1,040	130	–32	12	54	3.80
	Parietal	Frontal inferior operculum	45	R	2,608	326	56	10	22	3.92
		Inferior parietal lobule	40	L	920	115	–46	–46	46	3.48
	Limbic	Parahippocampal	–	R	640	80	14	–10	–18	3.34
		Cingulate gyrus	24	L	10,352	1294	–4	–8	50	4.07
	Cerebellum – posterior lobe	Declive Cerebellum and crus cerebri	N/A	L	904	113	–38	–60	–30	3.54
				R	1,016	127	28	–74	–28	3.77


*IS, Imagined slipping; BA, Broadmann area.*

**Table 2:** Differences in activation between imagined walking and rest conditions.

Contrast	Anatomical regions				mm^3^	Voxels	MNI coordinates			
	Lobe	Gyrus	BA	Side			*x*	*y*	*z*	Z-value
IW > Rest	Frontal	Middle frontal	32	L	808	101	–6	6	50	3.49


*IW, Imagined walking; BA, Broadmann area.*

### Mental Imagery of Slipping Versus Walking

**FIGURE 2:**
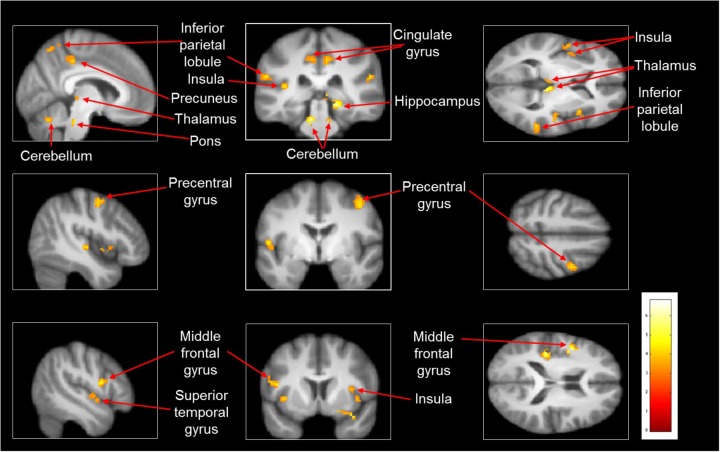
Heat map demonstrating areas with greater activation in imagined slipping versus imagined walking. Increased activation was noted in the precentral and middle frontal gyri, inferior parietal lobule (BA 40), precuneus, superior temporal gyrus (BA 22 and 38), parahippocampal gyrus, cingulate gyrus, pulvinar nucleus in thalamus, bilateral cerebellum, and pons in brainstem (*p*< 0.005).

Imagined slipping resulted in greater activation compared with imagined walking (**Table 3** and **Figure 2**). As compared with imagined walking, imagined slipping showed significantly more activation in left precentral and right middle frontal gyri, bilateral inferior parietal lobule (BA 40), precuneus, bilateral insula, right superior temporal gyrus (BA 22 and 38), right middle and left transverse temporal gyri, right parahippocampal gyrus, bilateral cingulate gyrus, right pulvinar nucleus in thalamus, right cerebellum, and pons in the brainstem.

**Table 3:** Differences in activation between imagined slipping and imagined walking.

Contrast	Anatomical regions				mm^3^	Voxels	MNI coordinates			
	Lobe	Gyrus	BA	Side			*x*	*y*	*z*	*Z*-value
IS > IW	Frontal	Precentral	–	L	704	88	–52	–4	6	3.38
		Middle frontal	–	R	968	121	42	–4	48	3.33
	Parietal	Inferior parietal lobule	40 40	R L	1,288 704	161 88	56 –56	–34 –30	24 24	3.32 3.41
		Precuneus	7		480	60	–8	–64	56	3.15
	Temporal	Superior temporal	38 22	R R	440 720	55 90	36 46	6 –18	–22 0	3.84 3.22
		Middle temporal	–	R	848	106	54	–42	0	3.79
		Transverse temporal	–	L	1,080	135	–38	–28	12	4.17
	Limbic	Parahippocampal Cingulate	35 31	R R L	1,056 984 768	132 123 96	18 8 –6	–30 –30 –34	–10 42 42	4.00 3.19 3.25
	Insula	Insula L Rolandic Oper Undefined	–	L L R	624 1,048 928	78 131 116	–40 –44 34	0 4 10	0 14 10	3.35 4.16 3.62

	Thalamus	Pulvinar	–	R	640	80	8	–28	2	4.01
	Brainstem	Pons	–	L	1,256	157	–8	–32	–24	3.85
	Cerebellum Anterior Lobe Posterior Lobe	Undefined Declive of vermis	– NA	R R	600 984	75 123	18 2	–42 –70	–30 –26	3.30 3.14


*IS, imagined slipping; IW, imagined walking; BA, Broadmann area.*

### Mental Imagery Versus Observation of Walking

The contrast comparing mental imagery of walking to observation of walking did not reveal any active voxels at the specified threshold. Thus, there was no difference in the areas of activation between mental imagery and observation of walking.

### Mental Imagery Versus Observation of Slipping

**FIGURE 3:**
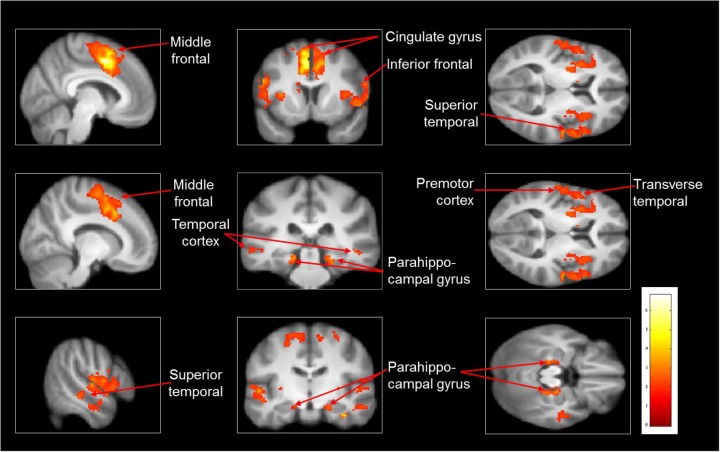
Heat map showing areas with increased activation in the imagined slipping compared with observed slipping. Additional activation was noted in supplementary motor area (BA 6), anterior prefrontal cortex (BA 10), superior temporal gyri, bilateral parahippocampal gyrus, cingulate gyrus, and inferior parietal lobule (*p*< 0.005) in imagined slipping versus observed slipping.

As compared with observed slipping, the mental imagery of slipping showed additional activation in left supplementary motor area (BA 6), bilateral middle frontal gyrus (BA 10), left superior temporal gyri, bilateral parahippocampal gyrus, left cingulate gyrus. (**Table 4** and **Figure 3**). Also, as opposed to observation of slipping, mental imagery of slipping yielded greater activation in the left inferior parietal lobule. Both mental imagery and observation of slipping showed similar activation in the inferior frontal gyrus and limbic regions.

**Table 4:** Differences in activation between imagined slipping and observed slipping.

Contrast	Anatomical regions				mm^3^	Voxels	MNI coordinates			
	Lobe	Gyrus	BA	Side			*x*	*y*	*z*	*Z*-value
IS > OS	Frontal	Middle frontal	–	R	2,768	346	36	32	26	4.28
			10	L	1,368	171	–36	40	24	3.46
		Inferior frontal	–	L	4,280	535	–36	30	2	3.85
	Parietal	Inferior Parietal lobule Extra Nuclear	40	R	536	67	64	–42	32	4.10
			–	L	624	78	–16	2	14	3.70
	Temporal	Superior temporal		L	9,584	1198	–52	–18	0	4.17
	Limbic	Parahippocampal	–	R	13,160	1645	36	–16	–28	4.46
				L	1,720	215	–16	–36	–12	3.95
				R	1,328	166	20	–20	–16	3.63
		Cingulate (SMA)	–	L	27,216	3402	–4	4	46	5.09


*IS: Imagined slipping, OS: Observed slipping, BA: Broadmann area, SMA: Supplementary motor areas.*

## Discussion

The purpose of this pilot study was to examine the neural correlates associated with slipping while walking. As hypothesized, activation of additional brain areas was seen during imagined slipping as compared with imagined walking. Our other major findings include increased activation during imagined slipping as compared with observed slipping and no additional activation in imagined walking as compared to observed walking.

### Mental Imagery of Slipping Versus Walking

The middle frontal gyrus was the common area activated between the imagined walk minus rest and imagined slip minus rest contrasts. However, imagined slip condition showed additional activation in areas such as supplementary motor area (SMA), dorsolateral prefrontal cortex, inferior parietal lobule, cerebellum, precuneus, cingulate gyrus, parahippocampal gyri and pulvinar nucleus of the thalamus over imagined walking. The brain activation pattern noted for imagined slipping was remarkably similar to previous studies which found activation in SMA, frontal cortex and cerebellum during imagery of dynamic balancing tasks (Ferraye et al., 2014; Taube et al., 2015).

The increased activation in the SMA during imagined slip condition over the imagined walk condition could be assumed as a response to perturbation causing activation of the SMA-cerebellar loop via the pontine nucleus and back to the motor cortex or association area in order to modify the ongoing movement. SMA, is mainly related to movement execution, motor planning and coordination along with dorsolateral prefrontal cortex, which help in response selection using prior associations (Eccles, 1982; Chouinard and Paus, 2006). These areas generate an appropriate motor response, including corrective motor responses, to prevent loss of balance or fall. Our findings are in line with EEG studies performed during standing perturbations, which indicated the presence of N1, negative event-related potential occurring about 100 ms after onset of an unpredictable stance perturbation in the fronto-central cortical sites (Quant et al., 2004; Adkin et al., 2006; Mochizuki et al., 2009). In these studies, small magnitude perturbations were delivered which evoked feet-in-place postural responses. In addition, it was found that the amplitude of the N1 response changed with the knowledge of an impending perturbation such that the N1 amplitude was greater during an unpredictable perturbation (Adkin et al., 2006). Similarly, Mihara et al. (2012) using fNIRS, examined cortical activation during anterior-posterior and medio-lateral stance perturbations in individuals with stroke. It was found that postural responses were accompanied by increased uptake of oxygenated hemoglobin in bilateral prefrontal, premotor and parietal cortex association areas. Thus, these studies suggest the role of frontal cortex in detection of balance loss and in modulating recovery responses (Dietz et al., 1984).

In order to correct movement error for restoring and maintaining the COM within the BOS, the cerebellum could be activated. Since cerebellum (vermis) acts as a comparator for signals received from the proprioceptors of the moving body segments via the spinocerebellar tract and timely modulate the response to match the intended movement, it could be involved in planning of the reactive stepping response (Pisotta and Molinari, 2014).

We found activation of areas related to visuo-spatial navigation such as the precuneus region of the parietal lobe and parahippocampal gyri. Activation in these regions could be related to the mental visualization of the slipping environment (Cavanna and Trimble, 2006) and formation of associations between visual and spatial information, thus helping in integration of the walking path (McNaughton et al., 1996). Furthermore, parahippocampal gyri could also be associated to the retrieval of past memory in order to generate the reactive balance responses (Ekstrom and Bookheimer, 2007).

Considering the unique sensory experience provided by slipping, the activation of inferior parietal lobule could be associated with the integration of somatosensory and visual stimuli (Singh-Curry and Husain, 2009). Similarly, activation of thalamus could be attributed to sensory integration, processing and maintaining erect posture while recovering balance from falls (Zwergal et al., 2009; Barra et al., 2010). Activation in insula which helps in perception, motor control and self-awareness was also noted (Sterzer and Kleinschmidt, 2010).

There was significant activation in the cingulate gyrus, which plays a role in monitoring error while performing motor tasks and could be of significance in detecting balance loss during walking (Holroyd et al., 2004; Debener et al., 2005; Anguera et al., 2009; Sipp et al., 2013). Our findings are similar to the results from a recent study, which used high-density EEG along with independent component analysis to identify cortical activity related to loss of balance (Sipp et al., 2013). The authors found that walking on a balance beam mounted on a treadmill was associated with activation of anterior cingulate, posterior cingulate, anterior parietal, sensorimotor and dorsolateral-prefrontal cortices (Sipp et al., 2013).

Despite some similarities in activations observed between imagined slipping and walking conditions, additional cortical and subcortical areas recruitment during imagined slipping suggest the involvement of different neural pathways in reactive balance control while walking than regular walking. This could be functionally or in real-life associated with the novelty of the sensorimotor stimulus and the perceived complexity of maintaining stability during a sudden slip while walking. It is established that longer-term training of a motor skill reduces the amount of cortical activation in motor areas suggesting a more efficient control of movement after sufficient practice (Jancke et al., 2000; Picard et al., 2013; Naito and Hirose, 2014). On similar lines, walking is a self-generated, anticipatory and a relatively learned (over years) task as compared with recovering balance during a sudden slip. Unlike regular walking, an unexpected disturbance during walking results in larger excursion of COM threatening postural stability. Such disturbance while walking might necessitate recruitment of a variable motor command than regular walking to control lower extremities and execute an appropriate postural response. It is thus likely that, slipping demands greater neural resources for sensory (visuo-spatial, perceptual, tactile, proprioceptive), cognitive and motor processing than regular walking, as that observed in our study.

### Mental Imagery Versus Observation of Walking

Neuroimaging studies have established the existence of a strong overlap in the neural areas activated during the actual task performance and its imagination and observation (Buccino et al., 2001; Grezes and Decety, 2002; Hanakawa et al., 2003). This evidence comes from studies examining brain activation during lower extremity movements (la Fougere et al., 2010; Dalla Volta et al., 2015). For example, Iseki et al. (2008) compared imagined and observed walking, and found that SMA and the dorsal premotor cortex (PMd) were activated in both the conditions (Iseki et al., 2008). Previous studies have shown that these two areas are essential for gait initiation, turning, negotiating obstacles and walking in narrow passages (Della Sala et al., 2002; Malouin et al., 2003; Nadeau, 2007). Another study examining neural activation during whole body gymnastic movements showed that similar motor areas were activated during both motor imagery and action observation (Munzert et al., 2008). Furthermore, Orr et al. (2008) observed that both imagination and observation of ankle movements was associated with common activation in inferior parietal lobule and precentral gyrus. Based on previous evidence, the fact that we did not observe any additional activation in imagined versus observed walking condition could be due to the common areas activated during these conditions.

### Mental Imagery Versus Observation of Slipping

Contrary to that seen for regular walking, for the slipping condition, our results suggest that there was additional activation noted during imagined versus the observed condition. Although motor imagery and observation could be driven by similar neural pathways (Grafton et al., 1996; Grezes and Decety, 2001; Jeannerod, 2001), it can be argued that first person motor imagery involves a more conscious brain engagement in an attempt to simulate the actual motor experience. This may be particularly important during tasks posing significant threat to balance and the CNS relies on activation of necessary visual-perceptual and motor systems to select an appropriate response. In our study, imagined slipping showed additional activation in SMA (BA 6) and bilateral middle frontal gyrus, which are involved in movement planning and initiation (Eccles, 1982; Chouinard and Paus, 2006); cingulate gyrus, which monitors motor error and detects balance loss during walking (Holroyd et al., 2004; Debener et al., 2005; Anguera et al., 2009; Sipp et al., 2013) and inferior frontal gyrus, which is involved in regulating excessive trunk movement to prevent loss of balance via response suppression (Sharp et al., 2010). Furthermore, there was recruitment of sensory and visual processing areas such as superior temporal gyri, bilateral parahippocampal gyrus and cingulate gyrus, (McNaughton et al., 1996; Yang et al., 2015) and areas related to perception and internal awareness such as inferior parietal lobule, resulting in increased activation (Singh-Curry and Husain, 2009). These findings clearly suggest greater engagement of neural structures while imagining a challenging locomotor-balance task like slipping. Our findings are also in agreement with the study by Taube et al. (2015) showing increased activation in SMA and the middle frontal gyrus during imagery over observation of maintaining standing balance on a wobble board which continuously disturbs balance. Thus, current and previous findings highlight that the extent of neural activation during motor imagery might also be influenced by task complexity, uniqueness or prior motor experience with the task.

## Clinical Application and Limitation

Considering that mental imagery showed greater activation of brain areas, it can be used to better examine the neural correlates than action observation for activities which cannot be overtly performed in the scanner. While a mental imagery paradigm is beneficial to evaluate gait related activities, the findings of this study should be interpreted in light of few limitations of this approach. There could be individual differences in the ability of the subjects to imagine even though no difference was noted on the scoring of the Vividness of motor and visual imagery questionnaire, which can alter the areas and the extent of clusters activated, as supported by van der Meulen et al. (2014). Furthermore, the quality of the imagined movement is dependent on the attentiveness during imagination (De Beni et al., 2007; Gregg et al., 2010). The elimination of action of gravity on the otherwise weight bearing activities of walking and slipping may have some effect on the overall activation pattern. Also, the observed differences between activations during mental imagery and rest or observed conditions could be due to the fact that the eyes were closed in the imagined condition and open during rest and observed conditions.

Since our study is a pilot study, the study may be underpowered owing to a smaller sample size. However, this pilot study does provide a proof of feasibility, and estimates of effect sizes, so that larger studies, needed to rule out type I error can be planned. To evaluate potential future clinical application of our results in other population, a larger sample size with variable hand dominance would be recommended. Further, examining those with known gait disturbances can also provide convergent validity for these results. In spite of these limitations, we had the advantage of better spatial resolution as compared with other imaging techniques provided by use of *fMRI*(Chen and Ugurbil, 1999).

The results of this study suggest that a network of cortical and subcortical areas somewhat different from regular walking is associated with the act of balancing in response to a novel slip. It further corroborates the view that specific cortical regions play a role in reactive balance control which has been proposed by several behavioral (Rankin et al., 2000; Brauer et al., 2002; Adkin et al., 2006) and animal studies (Beloozerova and Sirota, 1993; Deliagina et al., 2007). This study elucidates the neural structures associated with slipping in a young healthy nervous system. Future studies should investigate the brain activation patterns in elderly and in populations with balance deficits to further inform neural mechanisms of reactive balance control and potential causes of falls in these individuals.

## Disclosure

No commercial party having a direct financial interest in the research findings reported here has conferred or will confer a benefit on the authors or on any organization with which the authors are associated.

## Ethics Statement

A written informed consent was obtained from all research participants. The study protocol was approved by the Institutional Review at University of Illinois at Chicago.

## Author Contributions

TB provided the conceptual framework and experimental design, facilities, funding, and assistance with manuscript preparation. SL has contributed toward experimental design, data processing and analysis, consultation and reviewing of manuscript. PP and SD assisted with experimental design, data collection, analysis, and manuscript preparation. SRD has assisted with data processing, analysis, and manuscript review.

## Conflict of Interest Statement

The authors declare that the research was conducted in the absence of any commercial or financial relationships that could be construed as a potential conflict of interest.

## Footnotes

1 http://www.fil.ion.ucl.ac.uk/spm/

2 http://afni.nimh.nih.gov/afni/

3 http://fsl.fmrib.ox.ac.uk/fsl/fslwiki/

## References

[B1] Adkin A. L. Quant S. Maki B. E. McIlroy W. E. (2006). Cortical responses associated with predictable and unpredictable compensatory balance reactions. *Exp. Brain Res.* 172 85–93. 10.1007/s00221-005-0310-9 16418848

[B2] Allum J. Honegger F. (1992). A postural model of balance-correcting movement strategies. *J. Vestib. Res.* 2 323–347. 1342406

[B3] Anguera J. A. Seidler R. D. Gehring W. J. (2009). Changes in performance monitoring during sensorimotor adaptation. *J. Neurophysiol.* 102 1868–1879. 10.1152/jn.00063.2009 19605614 PMC2746769

[B4] Armstrong D. M. Drew T. (1985). Forelimb electromyographic responses to motor cortex stimulation during locomotion in the cat. *J. Physiol.* 367 327–351. 10.1113/jphysiol.1985.sp015827 4057102 PMC1193066

[B5] Aruin A. S. Latash M. L. (1995). The role of motor action in anticipatory postural adjustments studied with self-induced and externally triggered perturbations. *Exp. Brain Res.* 106 291–300. 10.1007/BF00241125 8566194

[B6] Barra J. Marquer A. Joassin R. Reymond C. Metge L. Chauvineau V. (2010). Humans use internal models to construct and update a sense of verticality. *Brain* 133(Pt 12), 3552–3563. 10.1093/brain/awq311 21097492

[B7] Beloozerova I. N. Sirota M. G. (1993). The role of the motor cortex in the control of accuracy of locomotor movements in the cat. *J. Physiol.* 461 1–25. 10.1113/jphysiol.1993.sp019498 8350259 PMC1175242

[B8] Berger W. Dietz V. Quintern J. (1984). Corrective reactions to stumbling in man: neuronal co-ordination of bilateral leg muscle activity during gait. *J. Physiol.* 357 109–125. 10.1113/jphysiol.1984.sp015492 6512687 PMC1193250

[B9] Bhatt T. Pai Y. C. (2008). Can observational training substitute motor training in preventing backward balance loss after an unexpected slip during walking? *J. Neurophysiol.* 99 843–852. 10.1152/jn.00720.2007 18003882 PMC2810608

[B10] Brauer S. G. Woollacott M. Shumway-Cook A. (2002). The influence of a concurrent cognitive task on the compensatory stepping response to a perturbation in balance-impaired and healthy elders. *Gait Posture* 15 83–93. 10.1016/S0966-6362(01)00163-1 11809584

[B11] Buccino G. (2014). Action observation treatment: a novel tool in neurorehabilitation. *Philos. Trans. R. Soc. Lond. B Biol. Sci.* 369:20130185. 10.1098/rstb.2013.0185 24778380 PMC4006186

[B12] Buccino G. Binkofski F. Fink G. R. Fadiga L. Fogassi L. Gallese V. (2001). Action observation activates premotor and parietal areas in a somatotopic manner: an fMRI study. *Eur. J. Neurosci.* 13 400–404. 10.1046/j.1460-9568.2001.01385.x 11168545

[B13] Caligiore D. Mustile M. Spalletta G. Baldassarre G. (2017). Action observation and motor imagery for rehabilitation in Parkinson’s disease: a systematic review and an integrative hypothesis. *Neurosci. Biobehav. Rev.* 72 210–222. 10.1016/j.neubiorev.2016.11.005 27865800

[B14] Cavanna A. E. Trimble M. R. (2006). The precuneus: a review of its functional anatomy and behavioural correlates. *Brain* 129(Pt 3), 564–583. 10.1093/brain/awl004 16399806

[B15] Chen W. Ugurbil K. (1999). High spatial resolution functional magnetic resonance imaging at very-high-magnetic field. *Top. Magn. Reson. Imaging* 10 63–78. 10.1097/00002142-199902000-00006 10389673

[B16] Chouinard P. A. Paus T. (2006). The primary motor and premotor areas of the human cerebral cortex. *Neuroscientist* 12 143–152. 10.1177/1073858405284255 16514011

[B17] Cox R. W. Chen G. Glen D. R. Reynolds R. C. Taylor P. A. (2017). FMRI clustering in AFNI: false-positive rates redux. *Brain Connect.* 7 152–171. 10.1089/brain.2016.0475 28398812 PMC5399747

[B18] Dalla Volta R. Fasano F. Cerasa A. Mangone G. Quattrone A. Buccino G. (2015). Walking indoors, walking outdoors: an fMRI study. *Front. Psychol.* 6:1502. 10.3389/fpsyg.2015.01502 26483745 PMC4589641

[B19] De Beni R. Pazzaglia F. Gardini S. (2007). The generation and maintenance of visual mental images: evidence from image type and aging. *Brain Cogn.* 63 271–278. 10.1016/j.bandc.2006.09.004 17074426

[B20] Debener S. Ullsperger M. Siegel M. Fiehler K. von Cramon D. Y. Engel A. K. (2005). Trial-by-trial coupling of concurrent electroencephalogram and functional magnetic resonance imaging identifies the dynamics of performance monitoring. *J. Neurosci.* 25 11730–11737. 10.1523/jneurosci.3286-05.2005 16354931 PMC6726024

[B21] Decety J. Jeannerod M. (1995). [Imagery and its neurological substrate]. *Rev. Neurol.* 151 474–479. 8578067

[B22] Deliagina T. G. Zelenin P. V. Beloozerova I. N. Orlovsky G. N. (2007). Nervous mechanisms controlling body posture. *Physiol. Behav.* 92 148–154. 10.1016/j.physbeh.2007.05.023 17561175

[B23] Della Sala S. Francescani A. Spinnler H. (2002). Gait apraxia after bilateral supplementary motor area lesion. *J. Neurol. Neurosurg. Psychiatry* 72 77–85. 10.1136/jnnp.72.1.77 11784830 PMC1737704

[B24] Diener H. C. Horak F. B. Nashner L. M. (1988). Influence of stimulus parameters on human postural responses. *J. Neurophysiol.* 59 1888–1905. 10.1152/jn.1988.59.6.1888 3404210

[B25] Dietz V. Quintern J. Berger W. (1984). Cerebral evoked potentials associated with the compensatory reactions following stance and gait perturbation. *Neurosci. Lett.* 50 181–186. 10.1016/0304-3940(84)90483-X 6493623

[B26] Dietz V. Quintern J. Boos G. Berger W. (1986). Obstruction of the swing phase during gait: phase-dependent bilateral leg muscle coordination. *Brain Res.* 384 166–169. 10.1016/0006-8993(86)91233-3 3790992

[B27] Drew T. (1988). Motor cortical cell discharge during voluntary gait modification. *Brain Res.* 457 181–187. 10.1016/0006-8993(88)90073-X 3167563

[B28] Eccles J. C. (1982). The initiation of voluntary movements by the supplementary motor area. *Arch. Psychiatr. Nervenkr.* 231 423–441. 10.1007/BF00342722 6812546

[B29] Edwards M. G. Humphreys G. W. Castiello U. (2003). Motor facilitation following action observation: a behavioural study in prehensile action. *Brain Cogn.* 53 495–502. 10.1016/S0278-2626(03)00210-0 14642300

[B30] Ekstrom A. D. Bookheimer S. Y. (2007). Spatial and temporal episodic memory retrieval recruit dissociable functional networks in the human brain. *Learn. Mem.* 14 645–654. 10.1101/lm.575107 17893237 PMC2044556

[B31] Eng J. J. Winter D. A. Patla A. E. (1994). Strategies for recovery from a trip in early and late swing during human walking. *Exp. Brain Res.* 102 339–349. 10.1007/BF00227520 7705511

[B32] Ferber R. Osternig L. R. Woollacott M. H. Wasielewski N. J. Lee J.-H. (2002). Reactive balance adjustments to unexpected perturbations during human walking. *Gait Posture* 16 238–248. 10.1016/S0966-6362(02)00010-3 12443948

[B33] Ferraye M. U. Debu B. Heil L. Carpenter M. Bloem B. R. Toni I. (2014). Using motor imagery to study the neural substrates of dynamic balance. *PLoS One* 9:e91183. 10.1371/journal.pone.0091183 24663383 PMC3963848

[B34] Forssberg H. Grillner S. Rossignol S. (1975). Phase dependent reflex reversal during walking in chronic spinal cats. *Brain Res.* 85 103–107. 10.1016/0006-8993(75)91013-6 1109686

[B35] Franceschini M. Agosti M. Cantagallo A. Sale P. Mancuso M. Buccino G. (2010). Mirror neurons: action observation treatment as a tool in stroke rehabilitation. *Eur. J. Phys. Rehab. Med.* 46 517–523. 20414184

[B36] Garcia Carrasco D. Aboitiz Cantalapiedra J. (2016). Effectiveness of motor imagery or mental practice in functional recovery after stroke: a systematic review. *Neurologia* 31 43–52. 10.1016/j.nrl.2013.02.003 23601759

[B37] Glover G. H. Thomason M. E. (2004). Improved combination of spiral-in/out images for BOLD fMRI. *Magn. Reson. Med.* 51 863–868. 10.1002/mrm.20016 15065263

[B38] Grafton S. T. Arbib M. A. Fadiga L. Rizzolatti G. (1996). Localization of grasp representations in humans by positron emission tomography. 2. Observation compared with imagination. *Exp. Brain Res.* 112 103–111. 10.1007/BF00227183 8951412

[B39] Graydon F. X. Friston K. J. Thomas C. G. Brooks V. B. Menon R. S. (2005). Learning-related fMRI activation associated with a rotational visuo-motor transformation. *Brain Res. Cogn. Brain Res.* 22 373–383. 10.1016/j.cogbrainres.2004.09.007 15722208

[B40] Gregg M. Hall C. Butler A. (2010). The MIQ-RS: a suitable option for examining movement imagery ability. *Evid. Based Complement. Alternat. Med.* 7 249–257. 10.1093/ecam/nem170 18955294 PMC2862926

[B41] Grezes J. Decety J. (2001). Functional anatomy of execution, mental simulation, observation, and verb generation of actions: a meta-analysis. *Hum. Brain Mapp.* 12 1–19. 10.1002/1097-0193(200101)12:1<1::AID-HBM10>3.0.CO;2-V 11198101 PMC6872039

[B42] Grezes J. Decety J. (2002). Does visual perception of object afford action? Evidence from a neuroimaging study. *Neuropsychologia* 40 212–222. 10.1016/S0028-3932(01)00089-6 11640943

[B43] Hanakawa T. Immisch I. Toma K. Dimyan M. A. Van Gelderen P. Hallett M. (2003). Functional properties of brain areas associated with motor execution and imagery. *J. Neurophysiol.* 89 989–1002. 10.1152/jn.00132.2002 12574475

[B44] Hardwick R. M. Edwards M. G. (2011). Observed reach trajectory influences executed reach kinematics in prehension. *Q. J. Exp. Psychol.* 64 1082–1093. 10.1080/17470218.2010.538068 21287427

[B45] Holroyd C. B. Nieuwenhuis S. Yeung N. Nystrom L. Mars R. B. Coles M. G. (2004). Dorsal anterior cingulate cortex shows fMRI response to internal and external error signals. *Nat. Neurosci.* 7 497–498. 10.1038/nn1238 15097995

[B46] Hong I. K. Choi J. B. Lee J. H. (2012). Cortical changes after mental imagery training combined with electromyography-triggered electrical stimulation in patients with chronic stroke. *Stroke* 43 2506–2509. 10.1161/strokeaha.112.663641 22798329

[B47] Horak F. B. (2006). Postural orientation and equilibrium: what do we need to know about neural control of balance to prevent falls? *Age Ageing* 35(Suppl. 2), ii7–ii11. 10.1093/ageing/afl077 16926210

[B48] Ietswaart M. Johnston M. Dijkerman H. C. Joice S. Scott C. L. MacWalter R. S. (2011). Mental practice with motor imagery in stroke recovery: randomized controlled trial of efficacy. *Brain* 134(Pt 5), 1373–1386. 10.1093/brain/awr077 21515905 PMC3097892

[B49] Iseki K. Hanakawa T. Shinozaki J. Nankaku M. Fukuyama H. (2008). Neural mechanisms involved in mental imagery and observation of gait. *Neuroimage* 41 1021–1031. 10.1016/j.neuroimage.2008.03.010 18450480

[B50] Jacobs J. V. Horak F. B. (2007). Cortical control of postural responses. *J. Neural Transm.* 114 1339–1348. 10.1007/s00702-007-0657-0 17393068 PMC4382099

[B51] Jahn K. Deutschlander A. Stephan T. Kalla R. Wiesmann M. Strupp M. (2008). Imaging human supraspinal locomotor centers in brainstem and cerebellum. *Neuroimage* 39 786–792. 10.1016/j.neuroimage.2007.09.047 18029199

[B52] Jahn K. Deutschlander A. Stephan T. Strupp M. Wiesmann M. Brandt T. (2004). Brain activation patterns during imagined stance and locomotion in functional magnetic resonance imaging. *Neuroimage* 22 1722–1731. 10.1016/j.neuroimage.2004.05.017 15275928

[B53] Jancke L. Shah N. J. Peters M. (2000). Cortical activations in primary and secondary motor areas for complex bimanual movements in professional pianists. *Brain Res. Cogn. Brain Res.* 10 177–183. 10.1016/S0926-6410(00)00028-8 10978706

[B54] Jeannerod M. (2001). Neural simulation of action: a unifying mechanism for motor cognition. *Neuroimage* 14(1 Pt 2), S103–S109. 10.1006/nimg.2001.0832 11373140

[B55] Jenkinson M. Bannister P. Brady M. Smith S. (2002). Improved optimization for the robust and accurate linear registration and motion correction of brain images. *Neuroimage* 17 825–841. 10.1006/nimg.2002.1132 12377157

[B56] la Fougere C. Zwergal A. Rominger A. Forster S. Fesl G. Dieterich M. (2010). Real versus imagined locomotion: a [18F]-FDG PET-fMRI comparison. *Neuroimage* 50 1589–1598. 10.1016/j.neuroimage.2009.12.060 20034578

[B57] Luukinen H. Herala M. Koski K. Honkanen R. Laippala P. Kivela S. L. (2000). Fracture risk associated with a fall according to type of fall among the elderly. *Osteoporos. Int.* 11 631–634. 10.1007/s001980070086 11069199

[B58] Maki B. E. McIlroy W. E. (1997). The role of limb movements in maintaining upright stance: the ”change-in-support” strategy. *Phys. Ther.* 77 488–507. 10.1093/ptj/77.5.488 9149760

[B59] Malouin F. Richards C. L. Jackson P. L. Dumas F. Doyon J. (2003). Brain activations during motor imagery of locomotor-related tasks: a PET study. *Hum. Brain Mapp.* 19 47–62. 10.1002/hbm.10103 12731103 PMC6872050

[B60] Marx E. Stephan T. Nolte A. Deutschländer A. Seelos K. C. Dieterich M. (2003). Eye closure in darkness animates sensory systems. *Neuroimage* 19 924–934. 10.1016/S1053-8119(03)00150-2 12880821

[B61] Massion J. (1994). Postural control system. *Curr. Opin. Neurobiol.* 4 877–887. 10.1016/0959-4388(94)90137-6 7888772

[B62] McNaughton B. L. Barnes C. A. Gerrard J. L. Gothard K. Jung M. W. Knierim J. J. (1996). Deciphering the hippocampal polyglot: the hippocampus as a path integration system. *J. Exp. Biol.* 199(Pt 1), 173–185. 8576689 10.1242/jeb.199.1.173

[B63] Mihara M. Miyai I. Hattori N. Hatakenaka M. Yagura H. Kawano T. (2012). Cortical control of postural balance in patients with hemiplegic stroke. *Neuroreport* 23 314–319. 10.1097/WNR.0b013e328351757b 22357394

[B64] Mochizuki G. Sibley K. M. Cheung H. J. Camilleri J. M. McIlroy W. E. (2009). Generalizability of perturbation-evoked cortical potentials: independence from sensory, motor and overall postural state. *Neurosci. Lett.* 451 40–44. 10.1016/j.neulet.2008.12.020 19110034

[B65] Morganti F. Gaggioli A. Castelnuovo G. Bulla D. Vettorello M. Riva G. (2003). The use of technology-supported mental imagery in neurological rehabilitation: a research protocol. *Cyberpsychol. Behav.* 6 421–427. 10.1089/109493103322278817 14511455

[B66] Mulder T. (2007). Motor imagery and action observation: cognitive tools for rehabilitation. *J. Neural Transm.* 114 1265–1278. 10.1007/s00702-007-0763-z 17579805 PMC2797860

[B67] Munzert J. Zentgraf K. Stark R. Vaitl D. (2008). Neural activation in cognitive motor processes: comparing motor imagery and observation of gymnastic movements. *Exp. Brain Res.* 188 437–444. 10.1007/s00221-008-1376-y 18425505

[B68] Nadeau S. E. (2007). Gait apraxia: further clues to localization. *Eur. Neurol.* 58 142–145. 10.1159/000104714 17622719

[B69] Naito E. Hirose S. (2014). Efficient foot motor control by Neymar’s brain. *Front. Hum. Neurosci.* 8:594. 10.3389/fnhum.2014.00594 25136312 PMC4118031

[B70] Nashner L. M. (1976). Adapting reflexes controlling the human posture. *Exp. Brain Res.* 26 59–72. 10.1007/BF00235249 964327

[B71] Nashner L. M. (1980). Balance adjustments of humans perturbed while walking. *J. Neurophysiol.* 44 650–664. 10.1152/jn.1980.44.4.650 7431045

[B72] Orr E. L. Lacourse M. G. Cohen M. J. Cramer S. C. (2008). Cortical activation during executed, imagined, and observed foot movements. *Neuroreport* 19 625–630. 10.1097/WNR.0b013e3282fbf9e0 18382275

[B73] Pai Y. C. Bhatt T. S. (2007). Repeated-slip training: an emerging paradigm for prevention of slip-related falls among older adults. *Phys. Ther.* 87 1478–1491. 10.2522/ptj.20060326 17712033 PMC2826275

[B74] Patel J. Qiu Q. Yarossi M. Merians A. Massood S. Tunik E. (2017). Exploring the impact of visual and movement based priming on a motor intervention in the acute phase post-stroke in persons with severe hemiparesis of the upper extremity. *Disabil. Rehabil.* 39 1515–1523. 10.1080/09638288.2016.1226419 27636200 PMC5355001

[B75] Pavol M. J. Pai Y. C. (2007). Deficient limb support is a major contributor to age differences in falling. *J. Biomech.* 40 1318–1325. 10.1016/j.jbiomech.2006.05.016 16876174 PMC2825182

[B76] Pavol M. J. Runtz E. F. Pai Y. C. (2004). Diminished stepping responses lead to a fall following a novel slip induced during a sit-to-stand. *Gait Posture* 20 154–162. 10.1016/j.gaitpost.2003.08.004 15336285

[B77] Petersen T. H. Willerslev-Olsen M. Conway B. A. Nielsen J. B. (2012). The motor cortex drives the muscles during walking in human subjects. *J. Physiol.* 590 2443–2452. 10.1113/jphysiol.2012.227397 22393252 PMC3424763

[B78] Picard N. Matsuzaka Y. Strick P. L. (2013). Extended practice of a motor skill is associated with reduced metabolic activity in M1. *Nat. Neurosci.* 16 1340–1347. 10.1038/nn.3477 23912947 PMC3757119

[B79] Pisotta I. Molinari M. (2014). Cerebellar contribution to feedforward control of locomotion. *Front. Hum. Neurosci.* 8:475. 10.3389/fnhum.2014.00475 25009490 PMC4069484

[B80] Porro C. A. Francescato M. P. Cettolo V. Diamond M. E. Baraldi P. Zuiani C. (1996). Primary motor and sensory cortex activation during motor performance and motor imagery: a functional magnetic resonance imaging study. *J. Neurosci.* 16 7688–7698. 10.1523/JNEUROSCI.16-23-07688.1996 8922425 PMC6579073

[B81] Quant S. Adkin A. L. Staines W. R. Maki B. E. McIlroy W. E. (2004). The effect of a concurrent cognitive task on cortical potentials evoked by unpredictable balance perturbations. *BMC Neurosci.* 5:18. 10.1186/1471-2202-5-18 15147586 PMC428574

[B82] Rankin J. K. Woollacott M. H. Shumway-Cook A. Brown L. A. (2000). Cognitive influence on postural stability: a neuromuscular analysis in young and older adults. *J. Gerontol. A Biol. Sci. Med. Sci.* 55 M112–M119. 10.1093/gerona/55.3.M112 10795721

[B83] Saimpont A. Lafleur M. F. Malouin F. Richards C. L. Doyon J. Jackson P. L. (2013). The comparison between motor imagery and verbal rehearsal on the learning of sequential movements. *Front. Hum. Neurosci.* 7:773. 10.3389/fnhum.2013.00773 24302905 PMC3831159

[B84] Sharp D. J. Bonnelle V. De Boissezon X. Beckmann C. F. James S. G. Patel M. C. (2010). Distinct frontal systems for response inhibition, attentional capture, and error processing. *Proc. Natl. Acad. Sci. U.S.A.* 107 6106–6111. 10.1073/pnas.1000175107 20220100 PMC2851908

[B85] Shumway-Cook A. Woollacott M. H. (2007). *Motor Control: Translating Research into Clinical Practice*. Philadelphia, PA: Lippincott Williams & Wilkins.

[B86] Singh-Curry V. Husain M. (2009). The functional role of the inferior parietal lobe in the dorsal and ventral stream dichotomy. *Neuropsychologia* 47 1434–1448. 10.1016/j.neuropsychologia.2008.11.033 19138694 PMC2697316

[B87] Sipp A. R. Gwin J. T. Makeig S. Ferris D. P. (2013). Loss of balance during balance beam walking elicits a multifocal theta band electrocortical response. *J. Neurophysiol.* 110 2050–2060. 10.1152/jn.00744.2012 23926037 PMC3841925

[B88] Sterzer P. Kleinschmidt A. (2010). Anterior insula activations in perceptual paradigms: often observed but barely understood. *Brain Struct. Funct.* 214 611–622. 10.1007/s00429-010-0252-2 20512379

[B89] Tang P.-F. Woollacott M. H. Chong R. K. (1998). Control of reactive balance adjustments in perturbed human walking: roles of proximal and distal postural muscle activity. *Exp. Brain Res.* 119 141–152. 10.1007/s002210050327 9535563

[B90] Taube W. Mouthon M. Leukel C. Hoogewoud H. M. Annoni J. M. Keller M. (2015). Brain activity during observation and motor imagery of different balance tasks: an fMRI study. *Cortex* 64 102–114. 10.1016/j.cortex.2014.09.022 25461711

[B91] van der Meulen M. Allali G. Rieger S. W. Assal F. Vuilleumier P. (2014). The influence of individual motor imagery ability on cerebral recruitment during gait imagery. *Hum. Brain Mapp.* 35 455–470. 10.1002/hbm.22192 23015531 PMC6869533

[B92] Wang J. Wai Y. Weng Y. Ng K. Huang Y. Z. Ying L. (2009). Functional MRI in the assessment of cortical activation during gait-related imaginary tasks. *J. Neural Transm.* 116 1087–1092. 10.1007/s00702-009-0269-y 19669694

[B93] Woollacott M. H. Tang P. F. (1997). Balance control during walking in the older adult: research and its implications. *Phys. Ther.* 77 646–660. 10.1093/ptj/77.6.646 9184689

[B94] Yang F. Bhatt T. Pai Y. -C. (2009). Role of stability and limb support in recovery against a fall following a novel slip induced in different daily activities. *J. Biomech.* 42 1903–1908. 10.1016/j.jbiomech.2009.05.009 19520372 PMC2753595

[B95] Yang F. Bhatt T. Pai Y.-C. (2011). Limits of recovery against slip-induced falls while walking. *J. Biomech.* 44 2607–2613. 10.1016/j.jbiomech.2011.08.018 21899844 PMC3390211

[B96] Yang Y. L. Deng H. X. Xing G. Y. Xia X. L. Li H. F. (2015). Brain functional network connectivity based on a visual task: visual information processing-related brain regions are significantly activated in the task state. *Neural Regen. Res.* 10 298–307. 10.4103/1673-5374.152386 25883631 PMC4392680

[B97] Zehr E. P. Komiyama T. Stein R. B. (1997). Cutaneous reflexes during human gait: electromyographic and kinematic responses to electrical stimulation. *J. Neurophysiol.* 77 3311–3325. 10.1152/jn.1997.77.6.3311 9212277

[B98] Zhu M.-H. Wang J. Gu X.-D. Shi M.-F. Zeng M. Wang C.-Y. (2015). Effect of action observation therapy on daily activities and motor recovery in stroke patients. *Int. J. Nurs. Sci.* 2 279–282. 10.1016/j.jstrokecerebrovasdis.2015.02.022 25851344

[B99] Zwergal A. Strupp M. Brandt T. Buttner-Ennever J. A. (2009). Parallel ascending vestibular pathways: anatomical localization and functional specialization. *Ann. N. Y. Acad. Sci.* 1164 51–59. 10.1111/j.1749-6632.2009.04461.x 19645880

